# Maternal omega-3 fatty acid intake during neurodevelopment does not affect pup behavior related to depression, novelty, or learning

**DOI:** 10.1186/s13104-018-3915-3

**Published:** 2018-11-15

**Authors:** Corey Jackson, Douglas W. Barrett, Jason Shumake, Elisa Gonzales, F. Gonzalez-Lima, Michelle A. Lane

**Affiliations:** 1 Nutrition and Foods Program, School of Family and Consumer Sciences, Texas State University, 601 University Dr., San Marcos, TX 78666 USA; 2 Department of Psychology, The University of Texas at Austin, 108 E. Dean Keeton Stop A8000, Austin, TX 78712 USA

**Keywords:** n-3 fatty acids, Depression, Novelty reactivity learning, Memory, Neurodevelopment

## Abstract

**Objective:**

Previously, we showed that consumption of a diet supplemented with omega-3 polyunsaturated fatty acids (n-3FAs) for two rounds of gestation and lactation increased the ability of rat dams to cope with stress when compared to dams that ingested a diet lacking n-3FAs. The objective of this study was to determine if the diets of these dams affected the behavior of their pups later in life. To isolate the neurodevelopmental effects of n-3FAs, pups from the second gestation were weaned to a diet adequate in n-3FAs. Pup testing began at 8 weeks of age and consisted of the forced swim, open field, and hole board tests to examine depression-related behavior, reaction to novelty, and learning and memory, respectively.

**Results:**

Given the considerable difference in the n-3FA content of the maternal diet, we expected a large effect size, however with the exception of rearing duration, maternal diet did not affect behavior in any of the tests conducted. These results suggest that maternal n-3FA supplementation during neurodevelopment likely does not affect offspring behavior when a diet adequate in n-3FA is provided post-weaning. Rather, we hypothesize that brain n-3FAs at the time of testing confer altered behavior and corroborate the need for additional research.

**Electronic supplementary material:**

The online version of this article (10.1186/s13104-018-3915-3) contains supplementary material, which is available to authorized users.

## Introduction

Fetuses and neonates depend on docosahexaenoic acid (DHA), an omega-3 polyunsaturated fatty acid **(**n-3FA), via the placenta or milk to meet neurodevelopmental demands [1]. Higher maternal seafood intake and serum n-3FA concentrations during human pregnancy and lactation are often associated with increased infant neurocognitive development [2, 3] and cognitive, attentional, and emotional benefits lasting into childhood [4–9]; however, much work, including meta-analyses, shows no effects [10–12].

Rodent studies support the relationship between n-3FA consumption and beneficial behavioral outcomes [13–21], but few isolate the function of n-3FAs prior to weaning, during neurodevelopment. Our objective was to fill this gap. We used dams consuming disparate diets either deficient in or supplemented with n-3FAs through two rounds of gestation and lactation. This deficient diet reduced brain n-3FAs in dams [22, 23] and neonates [17]. In our previous study, supplemented dams exhibited an increased ability to cope with stress when compared to deficient dams [24]. In the current study, pups from the dams used in [24] were weaned to a diet containing adequate n-3FA levels for 5 weeks prior to behavioral testing. Because n-3FA levels were altered solely in the maternal diet but not in the pup diet after weaning, we could isolate behaviors potentially altered by maternal n-3FA intake during gestation and lactation, a crucial neurodevelopmental period.

## Main text

### Methods

#### Animals and diets

N-3FA deficient or supplemented diets were administered to Long Evans dams through two cycles of pregnancy and lactation (see Additional file 1). Maternal diets were manufactured by Research Diets, Inc. (New Brunswick, NJ), based on AIN-93G. Dams in the “without n-3FAs” group consumed diets with 7% sunflower oil. The “with n-3FAs” group ingested diets with 7% menhaden oil, consisting of 14.2% eicosapentaenoic acid (EPA) and 10.3% DHA.

Two days after parturition, litters were culled to eight pups, four/sex, when possible. Pups were housed with their dams until weaning at 21 days. Male pups from the second gestation of dams in [24] were used. Pups were from separate litters. Pups were weaned to the standard facility diet containing 6.7% fat (w/w), adequate α-linolenic acid (0.2% [25]), and negligible EPA and DHA. The fatty acid composition is in [24]. To simplify terminology, pup group names reflect the dams’ n-3FA intake.

Pups were habituated to handling prior to behavioral testing. Testing began 5 weeks after weaning, and continued for 7 days. Pups were sacrificed by rapid decapitation following the conclusion of the behavioral work. Protocols were approved by the Institutional Animal Care and Use Committees of the University of Texas at Austin and Texas State University.

#### Behavioral tests

The forced swim test (FST) was conducted as in [24]. Rats were observed for 5 min on day 2. Immobility was assigned to stationary postures, moving only to stay afloat, for more than 3 s.

The open field test (OFT) was performed in two 10-min sessions over two consecutive days [24]. The first day represented a novel; the second day a familiar environment. An activity monitoring system (Med Associates Inc., St. Albans, VT) recorded frequency, duration, speed, location, and the path of movement. Behaviors measured included exploration, average velocity, average rearing duration, time in the center 38% of the field, and stereotypic time.

The hole board test (HBT) measured nose pokes into a board with a 4 × 4 array of holes 2 cm in diameter, inserted into the OFT apparatus. During each trial, the same four out of 16 holes were baited with a piece of cookie. Pups were exposed to the cookies before testing. Trials began with the first entry and ended after the last bait was consumed or 5 min elapsed. Infrared beams below the floor recorded nose pokes (entries) into each hole. Latency to complete each trial, novel entries, repeat entries, and total entries were recorded by the automated system. Rats experienced five trials/day for three consecutive days.

#### Statistical analyses

Data for all subjects is included in figures. Data are expressed in tabular form in Additional files 2, 3, 4, and 5 as mean ± SEM, mean differences and 95% confidence intervals of the mean differences (CI) for n = 10 deficient and n = 7 supplemented pups unless indicated. Mean differences due to maternal diet were calculated by subtracting the value for the n-3FA deficient group from the supplemented group; thus, positive values indicate supplemented > deficient. Mean differences over time were calculated by subtracting the first day of testing from the last. Levene’s tests for equality of variance determined if Welch’s or Student’s t-tests were appropriate for analysis of behavior in the FST and on isolated days in the OFT and HBT (Additional files 2, 4). Fisher’s exact test assessed the proportion of pups with zero vs. non-zero immobility in the FST. Linear Mixed-Effects Modeling determined the effect of maternal diet on behavior over time in the OFT and HBT (Additional files 3, 5). This modeling was repeated dropping one subject at a time to determine if that individual drove the outcome. Statistical analyses were performed using SPSS v. 24 (Chicago, IL). Differences were considered significant at *P* < 0.05. The expected power analysis was done using G*Power 3.1 [26].

### Results

**Fig. 1:**
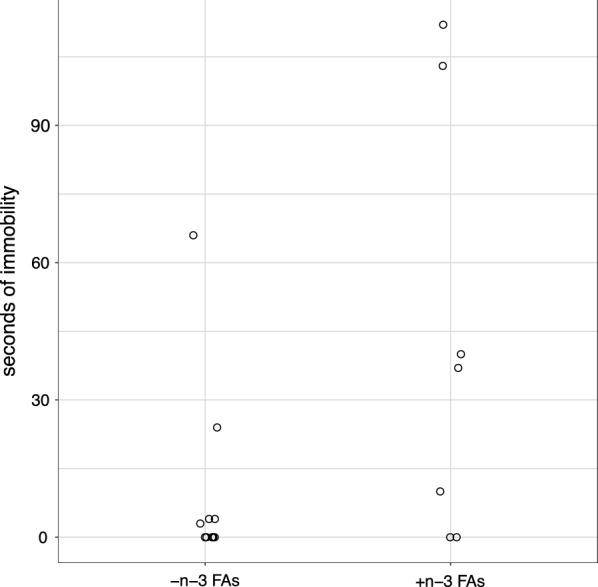
Effect of maternal n-3 FA consumption during neurodevelopment on offspring immobility in the forced swim test. Pups were placed in a cylinder of water, to simulate an inescapable stressor, for 15 min on day 1 and for 5 min on day 2. Day 2 video recordings were scored for immobility. Data shown are seconds of immobility on day 2 for pups with (+ n-3 FAs; n = 7) and without n-3 FAs during neurodevelopment (− n-3 FAs, n = 10)

Increased FST immobility characterizes depression-related behavior [27, 28]. There was no difference in the amount of time spent immobile due to maternal diet (Fig. 1), mean difference = 33.0 s, 95% CI (− 10.9, 77.0), *P* = 0.12. Maternal diet did not affect whether a pup displayed immobility (*P *= 0.62). Five pups per group exhibited immobility. Two supplemented and five deficient pups showed no immobility (Fig. 1).

**Fig. 2:**
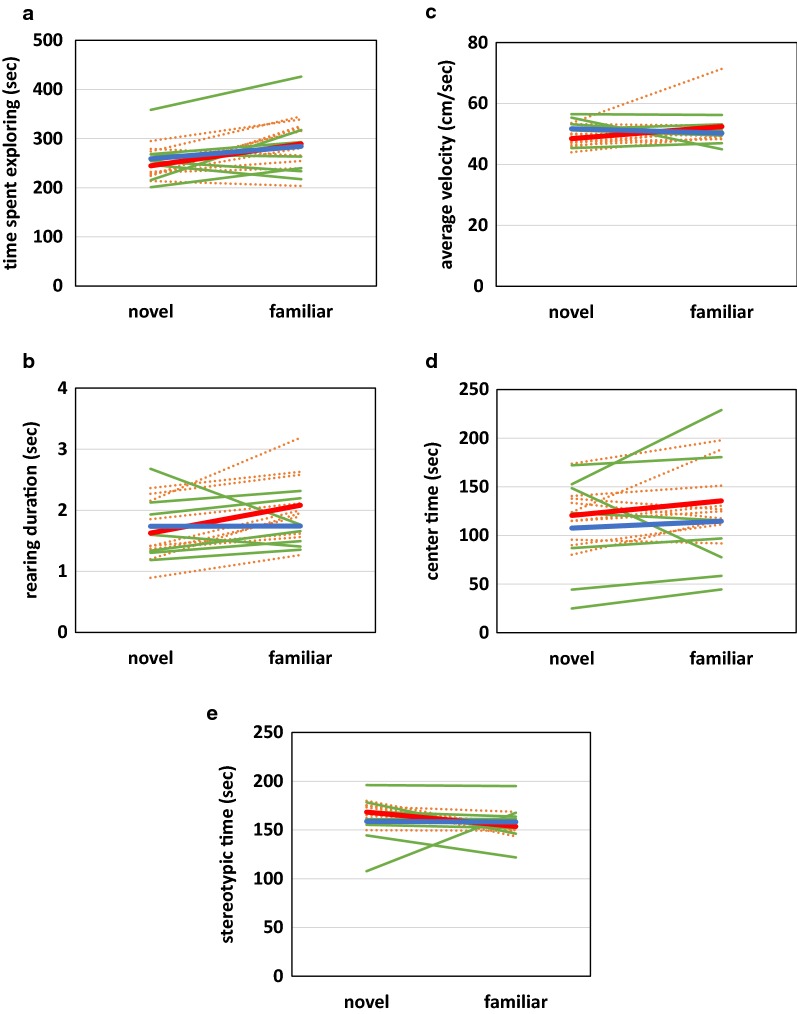
Impact of maternal n-3 FA consumption during neurodevelopment on offspring behavior in the open field. Pups were placed in the open field chamber for 10 min on day 1 (novel environment). Twenty-four hours later, pups were returned to the same chamber for another 10-min period (familiar environment). Individual trajectories in the novel and familiar open fields are shown for supplemented (n = 7) and deficient (n = 10) pups. In each panel, green, solid lines indicate supplemented pups and orange, dashed lines specify deficient pups. The means for supplemented pups are indicated by a thick, blue line. A thick, red line denotes the means for deficient pups. Panels show time spent exploring (**a**), average rearing duration (**b**), average velocity (**c**) center time (**d**) and stereotypic time (**e**)

Novelty reactivity predicts learned helplessness [29], predisposing to depression [30–32]. Group differences in activity changes between novel and familiar environments were assessed by comparing change in time spent exploring (the sum of ambulatory and rearing time), rearing duration average, and average velocity in the novel vs. familiar open field environments (Fig. 2; Additional file 2), which are reflected by the interaction between diet and time when predicting these metrics (Additional file 3). There was no effect of maternal diet on any parameter (Additional file 2). When modeled as a whole using linear mixed effects regression taking both maternal diet and day into account, diet did not alter time spent exploring, rearing duration, or average velocity (Fig. 2a–c; Additional file 3). Familiarity increased time spent exploring and rearing duration. A diet by novelty interaction existed for rearing duration. The rearing duration of supplemented pups remained relatively constant, while deficient pups increased their rearing duration between days (Additional files 2, 3). Due to the lack of response to maternal n-3FA intake on other parameters predictive of novelty response we believe this interaction is spurious and that, overall, n-3FA consumption during neurodevelopment has no effect on novelty reactivity.

Increased time spent in the center of the OFT environment reflects decreased fear, elevated risk-taking, or a combination thereof. Stereotypic movement signifies hyperactivity [33]. There were no effects of maternal diet or novelty on center or stereotypic time and no interactions (Fig. 2d, e; Additional files 2, 3).

**Fig. 3:**
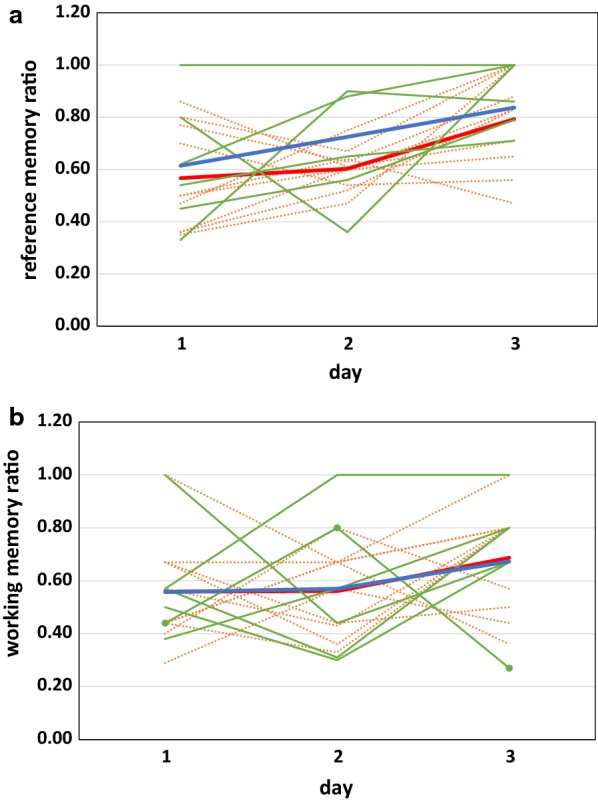
Consequences of maternal n-3 FA consumption on offspring reference or working memory in the hole board test. Pups were placed in the open field chamber modified to contain a board with a 4 × 4 grid and a consistent pattern of baited holes. Rats were subjected to five trials each day for three consecutive days. Individual trajectories are shown for n-3FA supplemented (n = 7) and deficient (n = 10) pups. In each panel, green, solid lines indicate supplemented pups and orange, dashed lines specify deficient pups. Solid, circular markers indicate the supplemented pup that was an outlier and not included in the working memory ratio data shown in Additional files 4 and 5. The mean memory ratios for supplemented pups is indicated by a thick, blue line. A thick, red line denotes the mean memory ratios for deficient pups. **a** Displays reference memory ratios, **b** working memory ratios. One less supplemented pup is included on day 2 for each ratio because data was lost due to an equipment malfunction

The HBT assessed learning and memory. The reference memory ratio equaled the number of entries to baited holes divided by the number of total entries (to both baited and unbaited holes). Maternal diet had no effect on reference memory ratios when each day of the HBT was considered separately (Additional file 4). Pups improved their performance across days but maternal diet had no effect on pup performance (Fig. 3a; Additional file 5). There was no interaction between maternal diet and day.

The working memory ratio equaled the number of initial entries to holes divided by the total number of entries plus re-entries to holes. Maternal diet did not affect working memory ratio. Elimination of one subject resulted in an increase in working memory ratio over time (Fig. 3b, Additional file 5). With this pup included the working memory ratio did not change over time [F (1,15) = 1.90, P-value = 0.17, mean difference = 0.12, 95% CI of mean difference (0.01, 0.24)]. There was no maternal diet by day interaction for working memory.

### Discussion

When subjects themselves ingest increased amounts n-3FAs studies often reveal a positive correlation between n-3FA intake and beneficial behavior. While many designs aim to examine the role of n-3FAs in neurodevelopment, few achieve this because the pups consume the same diet as their dams after weaning through testing. In the current design, dams consumed disparate levels of n-3FAs during gestation and lactation, but their pups ingested adequate n-3FAs from weaning onward, allowing isolation of behaviors perpetually altered by maternal n-3FA intake during neurodevelopment. We found no effect of n-3FA intake on behaviors related to depression, novelty reactivity, and learning and memory.

Others have shown an inverse relationship between depression-related behavior and n-3FA intake in adult rats [13, 34–42]. Those isolating neurodevelopment [21, 43] found that n-3FAs deficiency increased FST immobility in animals replete with n-3FAs post-weaning. Of note, diet did not affect depression-related behavior in the dams of our pups [24]. While we cannot be certain why these discrepancies exist, our use of Long-Evans (versus Sprague–Dawley) rats may contribute as strain affects FST behavior [44].

Studies indicate that as n-3FA intake increases, novelty reactivity decreases whether n-3FAs are consumed post-weaning [45] or from conception [17]. Post-weaning n-3FA supplementation also improves learning and memory in rats exposed to adequate n-3FA levels during neurodevelopment [34, 46]. Additionally, n-3FA deficiency during and after neurodevelopment decreases learning and brain DHA [18, 20]. In contrast to our study, in each of the above-mentioned studies n-3FA intake varied at the time of testing; thus, we hypothesize that the effects of n-3FAs on behavior are due to n-3FA levels in the brain during testing.

In conclusion, our work suggests that maternal n-3FA intake during neurodevelopment may not dramatically alter behavior later in life provided offspring n-3FA intake is adequate postweaning. At behavioral testing, all pups had consumed a diet with adequate n-3FA levels for 5 weeks, theoretically eliminating the effects of disparate maternal diets, resulting in similar brain n-3FA content as in [21, 43]. Additional work is required to test this theory.

## Limitations

### Sample size

Studies comparing diets either deficient in or supplemented with n-3FAs to those containing adequate levels have seen very large effect sizes with sample sizes of 6, 10, and 16 per group [14, 16, 18]. Based on these and considering the extreme disparity in n-3FA levels in the maternal diets, we chose n = 10/group for both generations. Indeed, we observed very large (Cohen’s d = 1.2) or large (Cohen’s d = 0.8–0.95) effect sizes of diet on dam behavior [24]. Unfortunately, three pups did not complete the study due to factors unrelated to diet. Our final sample size had an expected power of 62% to detect very large (Cohen’s d = 1.2) mean differences. Button et al. [47] showed that the median power of published neuroscience studies is 21%. Of note, studies with such low power can only obtain *P* < 0.05 significant differences if they randomly draw samples that show a much larger mean difference than the expected true effect. The fact that the vast majority of these publications (instead of the expected 21%) report significant differences reflects publication bias because underpowered studies with positive findings are published while underpowered studies with negative findings are often not. Thus, we conclude that our work was moderately well powered if the effect sizes of similar published studies are accurate estimates of the true effects; else, these previous studies are likely underpowered themselves and are therefore reporting exaggerated effects. Either way, it is important that our current negative findings are taken into account by future research, which should not assume very large effect sizes of n-3FA on behavior when calculating needed sample sizes. In summary, maternal n-3FA intake during neurodevelopment may not markedly effect behavior later in life, but we acknowledge this data does not prove the null and there could be effects of maternal n-3FA intake on offspring behavior that may be detected with a larger sample size.

### Lack of brain n-3FA analysis

The brains were used for other assays so their n-3FA content was not assessed.

## Additional files

**Additional file 1.** Experimental timeline.

**Additional file 2.** Table of activity parameters in the novel versus familiar open field.

**Additional file 3.** Table showing results of linear mixed effects regression analysis of open field activity parameters.

**Additional file 4.** Table of memory ratios by day in the hole board test.

**Additional file 5.** Table displaying results of linear mixed effects regression analysis of reference and working memory.

## Abbreviations

n-3FAs: omega-3 polyunsaturated fatty acids
DHA: docosahexaenoic acid
EPA: eicosapentaenoic acid
FST: forced swim test
OFT: open field test
HBT: hole board test
