# Supplementary material for: Maternal omega-3 fatty acid intake during neurodevelopment does not affect pup behavior related to depression, novelty, or learning
Source: BMC Res Notes. 2018 Nov 15;11:812. doi: 10.1186/s13104-018-3915-3 (PMC6238316; doi:10.1186/s13104-018-3915-3)
Supplement: Supplementary file 1 — Additional file 1. Experimental timeline. [file 13104_2018_3915_MOESM1_ESM.docx]

**Additional file 1.** Experimental timeline. The shaded boxes at the top reflect the period of time when the experimental diets, either supplemented or deficient in n-3 FAs, were consumed by dams during two cycles of gestation and lactation. Pups were housed with dams while nursing. Pups from the second gestation were separated from the dams and weaned onto the standard facility rodent diet, containing adequate n-3 FA levels, on day 21. All pups continued to consume this diet for 5 weeks until behavioral testing at 8 weeks of age. Behavioral testing began on day 1 of week 8, and lasted for 7 days.
